# Increased capsaicin receptor TRPV1 in skin nerve fibres and related vanilloid receptors TRPV3 and TRPV4 in keratinocytes in human breast pain

**DOI:** 10.1186/1472-6874-5-2

**Published:** 2005-03-08

**Authors:** Preethi Gopinath, Elaine Wan, Anita Holdcroft, Paul Facer, John B Davis, Graham D Smith, Chas Bountra, Praveen Anand

**Affiliations:** 1 Peripheral Neuropathy Unit, Hammersmith Hospital, Faculty of Medicine, Imperial College London, London, UK; 2 Department of Anaesthesia, Chelsea and Westminster Hospital, Faculty of Medicine, Imperial College London, London, UK; 3 Neurology CEDD, GlaxoSmithKline, Harlow, UK

## Abstract

**Background:**

Breast pain and tenderness affects 70% of women at some time. These symptoms have been attributed to stretching of the nerves with increase in breast size, but tissue mechanisms are poorly understood.

**Methods:**

Eighteen patients (n = 12 breast reduction and n = 6 breast reconstruction) were recruited and assessed for breast pain by clinical questionnaire. Breast skin biopsies from each patient were examined using immunohistological methods with specific antibodies to the capsaicin receptor TRPV1, related vanilloid thermoreceptors TRPV3 and TRPV4, and nerve growth factor (NGF).

**Results:**

TRPV1-positive intra-epidermal nerve fibres were significantly increased in patients with breast pain and tenderness (TRPV1 fibres / mm epidermis, median [range] – no pain group, n = 8, 0.69 [0–1.27]; pain group, n = 10, 2.15 [0.77–4.38]; p = 0.0009). Nerve Growth Factor, which up-regulates TRPV1 and induces nerve sprouting, was present basal keratinocytes: some breast pain specimens also showed NGF staining in supra-basal keratinocytes. TRPV4-immunoreactive fibres were present in sub-epidermis but not significantly changed in painful breast tissue. Both TRPV3 and TRPV4 were significantly increased in keratinocytes in breast pain tissues; TRPV3, median [range] – no pain group, n = 6, 0.75 [0–2]; pain group, n = 11, 2 [1-3], p = 0.008; TRPV4, median [range] – no pain group, n = 6, [0–1]; pain group, n = 11, 1 [0.5–2], p = 0.014).

**Conclusion:**

Increased TRPV1 intra-epidermal nerve fibres could represent collateral sprouts, or re-innervation following nerve stretch and damage by polymodal nociceptors. Selective TRPV1-blockers may provide new therapy in breast pain. The role of TRPV3 and TRPV4 changes in keratinocytes deserve further study.

## Background

Breast pain is a common problem, which can affect up to 70% of women [1]. Breast pain or mastalgia can be cyclical or non-cyclical. The cyclical type of breast pain has been attributed to sex hormonal changes through the menstrual cycle that may increase the size of the breast tissue, which stretches the internal structures and causes pain or soreness. Numerous studies have demonstrated variation in pain perception during the menstrual cycle [2-5]. Heat sensitivity is increased in the luteal (17–22) phase of the menstrual cycle [6] and lowest in the periovulatory phase (day 12–16), but other studies have shown variation at other times in the cycle. Non-cyclical breast pain can be caused by hormonal influences particularly oestrogen, and other causes such as macromastia, local infection or inflammation; rarely, breast cancer can present as breast pain. Macromastia may cause areas of numbness in the breast and problems with nipple erectile function, which is thought to be related to the stretching of the nerve supply with increase in breast size [7]. Post-surgical breast pain is also a significant entity, with about 50% of women who undergo mastectomy suffering from chronic pain one year after their operation [8,9].

The mechanisms of breast pain in the majority of women are not well understood at the cellular or molecular level. We hypothesized a relationship between clinical breast pain, nerve growth factor (NGF) and its regulated ion channels or receptors expressed by nociceptor fibres. Estrogens upregulate NGF receptor mRNA in sensory neurons [10], and enhance the proliferative effects of NGF [11,12]. As NGF is a key molecule that determines the sensitivity of nociceptors in humans [13] and animal models [14], sex hormonal influences could be responsible for altered NGF activity during the menstrual cycle, leading to cyclical breast soreness or pain. NGF expression is also increased by inflammation, and this is responsible for the collateral nerve fibre sprouting and hypersensitivity of nociceptor fibres associated with inflammation. The hypersensitivity is, in part, mediated via the capsaicin or vanilloid receptor 1 (TRPV1), which is required for thermal hyperalgesia in rodents [15,16], and is activated by heat pain. Thermal hyperalgesia can occur during the menstrual cycle and it is well known that the core body temperature alters during the cycle (this is a qualitative test for ovulation), and thus heat conductance and perception and tolerance of heat alters during the cycle [2,6]. The TRPV1 receptor is activated also by the products of inflammation. We have therefore studied TRPV1-expressing nerve fibres and NGF in skin from women with and without breast pain and tenderness. The recently discovered vanilloid thermoreceptors TRPV3 and TRPV4, which are also expressed by sensory fibres and activated by warmth, were also studied [17,18].

## Methods

### Patients

Eighteen patients were recruited (n = 12 breast reduction for macromastia; n = 6 breast reconstruction) at Chelsea and Westminster, Charing Cross, Ravenscourt Park Hospitals in London and Broomfield Hospital in Essex were recruited. Breast reduction patients had no previous surgery. The breast reconstruction patients had Latissimus dorsi flap reconstructions after previous mastectomies, and had implants. Patients below 18 years or above 70 years, with any local skin inflammation, infection or cancerous skin changes were excluded. The Research Ethics Committee of Hammersmith Hospitals Trust and Mid Essex Hospitals Trust gave ethical permission for the study. Informed consent was obtained prior to the clinical examination and questionnaire administration.

#### Clinical pain assessment

Age, parity, height, weight and menstrual data were collected. Details of current surgery, any previous breast surgery and breast disease were also recorded. A questionnaire which included questions on breast and period pain was administered, a diagram to indicate painful and tender areas and the 78 pain descriptors from the McGill Pain Questionnaire [19] were produced, along with a 10 cm unmarked visual analogue scale (VAS). The presence of breast pain was defined from the results of the Breast Pain Questionnaire using the total Pain Rating Index (PRI (total)) [19] and VAS scores marked by the patient in centimetres being more than zero to identify those patients with breast pain. Only two patients were taking simple analgesia for breast pain.

### Immunohistochemistry

Full thickness skin biopsies were collected from each patient along the incision line of about 2 mm depth. Samples were coded, frozen on site and stored at -70°C. The skin samples were mounted in embedding medium (Tissue-Tek OCT compound, Sakura Finetek, USA). Frozen tissue sections (10 μm) were collected onto poly-L-lysine-coated (Sigma Poole Dorset UK) glass slides and post-fixed in freshly prepared, 4% w/v paraformaldehyde in phosphate buffered saline (PBS; 0.1 M phosphate; 0.9% w/v saline; pH 7.3). After washing in PBS, endogenous peroxidase was blocked by incubation with 0.3% w/v hydrogen peroxide in methanol. After a further wash in PBS the tissue sections were incubated overnight with affinity purified antibodies to TRPV1 (polyclonal rabbit anti-TRPV1; GlaxoSmithKline, Harlow, UK; 1/5000; 1/10,000), TRPV3 (polyclonal rabbit anti-TRPV3; GlaxoSmithKline, Harlow, UK; 1/1000), TRPV4 (polyclonal rabbit anti-TRPV4; GlaxoSmithKline, Harlow, UK; 1/250; 1/1000), recombinant human NGF (polyclonal rabbit anti-NGF; Genentech, San Francisco, USA; 1/4000) or marker of large and some small calibre nerve fibres (mixed mouse monoclonal antibodies to neurofilaments 200 kD, 70 kD and 57 kD; DAKO cytomation Cambs., UK, 1/50,000; Novocastra, Newcastle upon Tyne, UK, 1/500). Methodological controls included omission of primary antibodies, or their replacement with pre-immune serum. Specificity of antibodies has been described in previous publications [18,20]. Sites of antibody attachment were revealed using biotinylated goat anti- rabbit or biotinylated horse anti-mouse IgG (Vector Laboratories, High Wycombe, Bucks., U.K.) and nickel-enhanced, immunoperoxidase (avidin-biotin complex – ABC elite; Vector Laboratories, High Wycombe, Bucks., U.K.). Nuclei were counterstained with 0.1% w/v aqueous neutral red. The intensity of NGF immunostaining was graded on a scale 0 – 3 where 0 = negative or no immunoproduct, 1 = weak immunoproduct, 2 = intermediate intensity immunoproduct and 3 = intense immunoproduct. Intra-epidermal and sub-epidermal TRPV1-, TRPV4 or neurofilament – positive fibres were counted and the length and thickness of the epidermis was measured using a calibrated microscope, eyepiece graticule. Similarly, fibres that extended through the epidermis were counted, along with arborising "clusters" of fibres. The two observers who performed the histological studies were blinded with regard to clinical pain scores.

### Statistical analyses

A non-parametric, two tailed test (Mann Whitney U) was used. Commercially available statistical software was used to perform the test (Prism 3™).

## Results

### Clinical pain assessment

Pain Rating Indices (PRI) and Visual Analogue Scores (VAS) were used to group patients with (n = 10) and without (n = 8) breast pain: pain group; PRI -Median (range) 12 (4–30); Mean (SEM) 13.44(3.07) and VAS – Median (range) 5(3.7–6.7); Mean (SEM) 5.14(0.31)]; in patients without breast pain, PRI and VAS were zero. Only 2 patients reported thermal pain descriptors (burning, hot), while most reported ache and tenderness. The numbers of breast reduction and reconstruction patients with pain were 7 out of 12 and 3 out of 6 respectively, and all reported pain of duration greater than 6 months. The presence (n = 8) or absence (n = 10) of dysmenorrhoea was also recorded. There was no association between breast pain and the presence of dysmenorrhoea.

### Immunostaining

#### TRPV1

**Figure 1:**
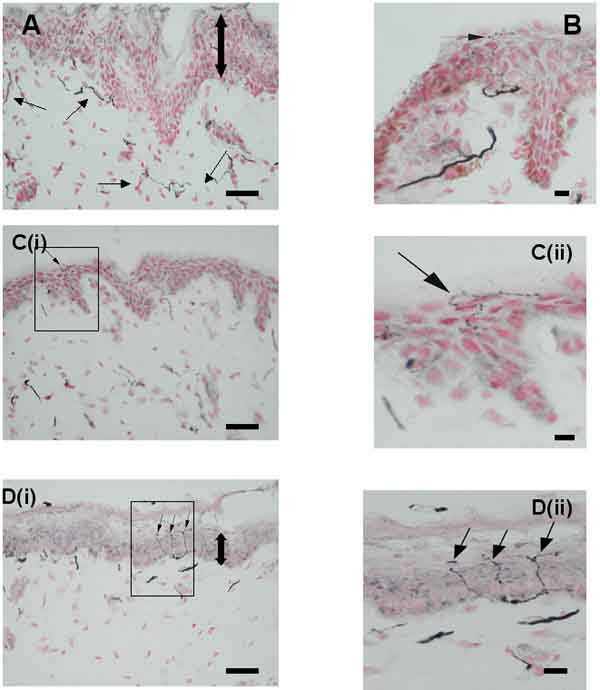
**TRPV1-immunoreactive nerve fibres in breast skin**. (**A**) Normal, control skin : TRPV1-immunoreactive nerve fibres (small arrows) in the sub-epidermis. (**B**) Painful skin (macromastia patient): intra-epidermal, TRPV1-immunoreactive nerve fibre (arrow) deriving from a large, sub-epidermal fascicle and extending to the stratum corneum. (**Ci**) Painful skin (breast reconstruction patient): TRPV1-immunoreactive intra-epidermal fibres passing along the junction between the epidermis and stratum corneum (**Cii**-enlarged area from **Ci**). (**Di**) Painful skin (macromastia patient): multiple branching, TRPV1-immunoreactive intra-epidermal nerve fibres (arrows) extending to the stratum corneum (**Dii**-enlarged area from **Di**). Large double arrows indicate relative epidermal thickness. Scale bars: A, B, C(i), D(i) = 50 μm; C(ii), D(ii) = 10 μm.

**Figure 2:**
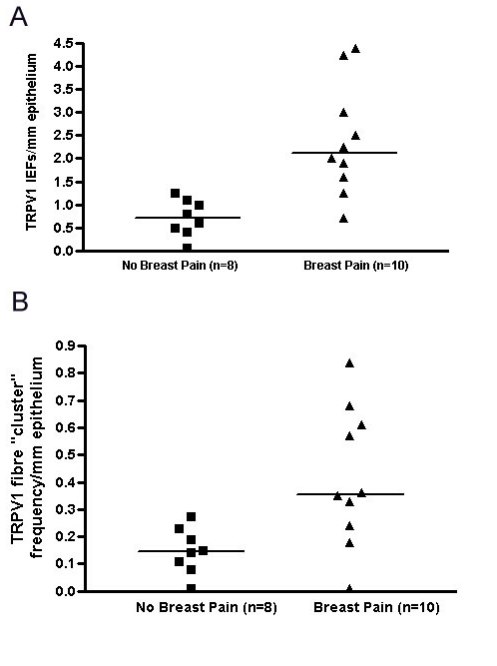
**Quantification of TRPV1-immunoreactive, intra-epidermal fibres and breast pain**. Scattergrams show TRPV1-immunoreactivity in (**A**) intra-epidermal fibres and (**B**) intra-epidermal fibre "clusters" in patients with and without breast pain.

TRPV1-immunoreactive fibres were present mainly in the sub-epidermis in normal (pain free) skin (Fig. 1A). In breast pain, TRPV1-immunoreactive fibres appeared to be more abundant in the epidermis and frequently seen to pass along the junction of the stratum corneum (intra-epidermal fibres – IEF; Fig. 1B, Ci, ii) often with multiple fibres (fibre "clusters"; Fig. 1Di, ii- arrows). Intra-epidermal fibres and fibre "cluster" counts/millimetre of epithelium were significantly higher in patients with breast pain (Table 1; Fig. 2). This significance was maintained despite exclusion of patients with previous breast surgery (i.e. the breast reconstruction patients; Pain, n = 7; No Pain, n = 5; p = 0.0303). While some specimens from pain patients showed thinning of the epidermis, this was not so overall for the pain group (Table 1).

**Table 1:** Histology results

	**Patients with no breast pain (N = 8)**	**Patients with breast pain (N = 10)**	**P-Mann Whitney Test**
**Intra-epidermal**	**Median (range)**	**Median (range)**	
TRPV1 intra-epidermal fibres	0.69 (0.00 – 1.27)	2.15 (0.77 – 4.38)	0.0009**
TRPVR1 fibre "clusters"	0.15 (0.00 – 0.27)	0.37 (0.00–0.85)	0.0085*
Sub-epidermal			
TRPV1 fibres	2.98 (1.59–4.04)	3.79 (0.63 – 5.92)	0.1457
Neurofilament fibres	3.38 (0.83 – 5.92)	2.95 (0.94 – 4.95)	0.7618
NGF staining	2.5 (1.0–3.0)	2.38 (1.0–3.0)	0.5726
Epidermal Thickness (mm)	0.04 (0.02 – 0.12)	0.04 (0.03 – 0.05)	0.9654

#### TRPV3

**Figure 3:**
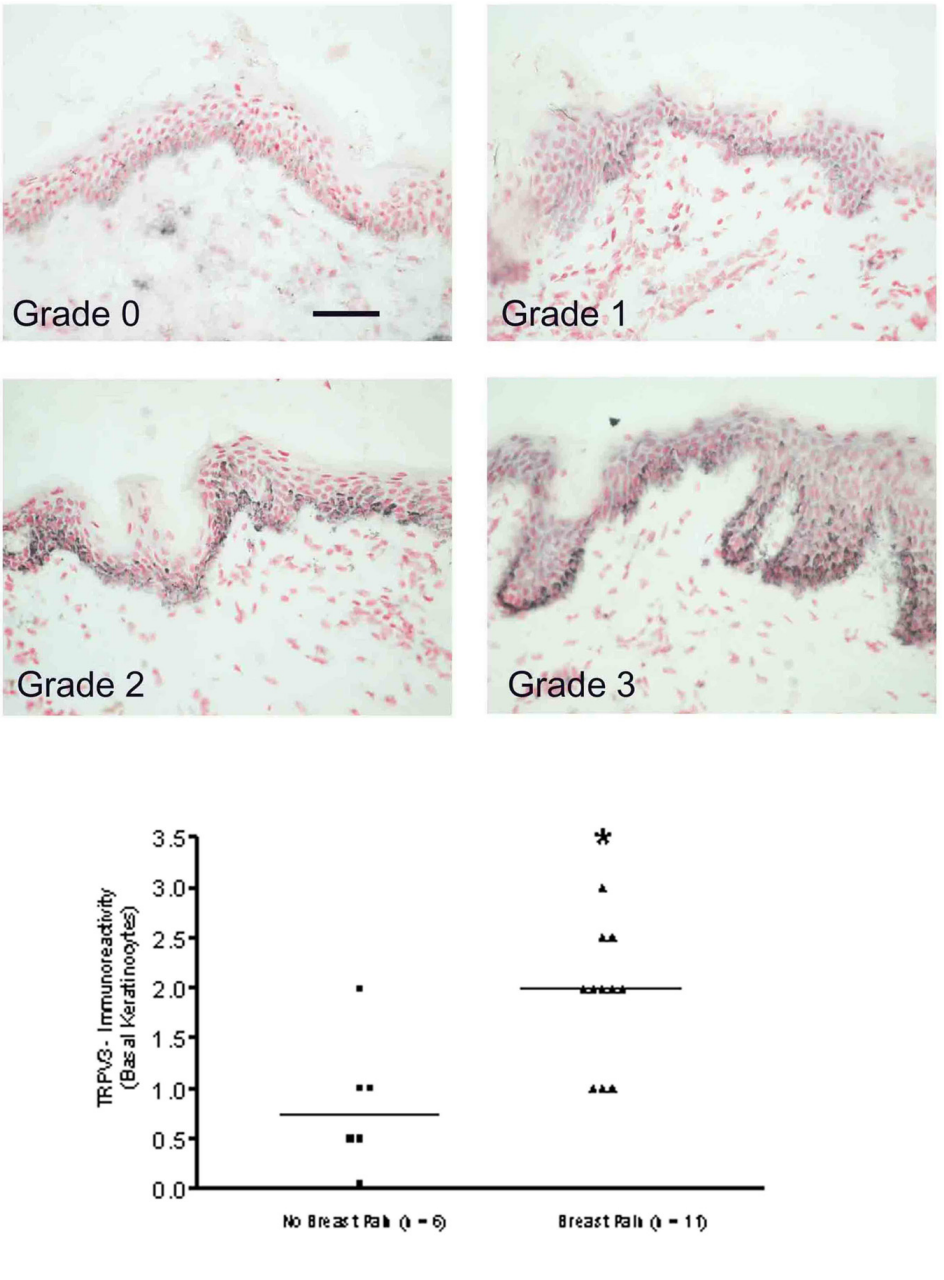
**TRPV3-immunoreactivity in breast skin**. TOP PANELS: TRPV3-immunoreactive keratinocytes mostly in basal layer and graded from grade 0/negative (top left) to grade 3/strong staining (bottom right). Scale bar = 100 μm BOTTOM PANEL: Scattergram showing grading assessment and a significant increase (*P < 0.05) of TRPV3 immunoreactivity in patients with pain.

TRPV3 immunoreactivity was detected in basal keratinocytes and occasional suprabasal cells throughout the epidermis. Quantification of immunostaining showed a significant (*P = 0.011) increase with breast pain (Fig. 3). TRPV3-immunoreactivity was not detected in skin nerve fibres in this study.

#### TRPV4

**Figure 4:**
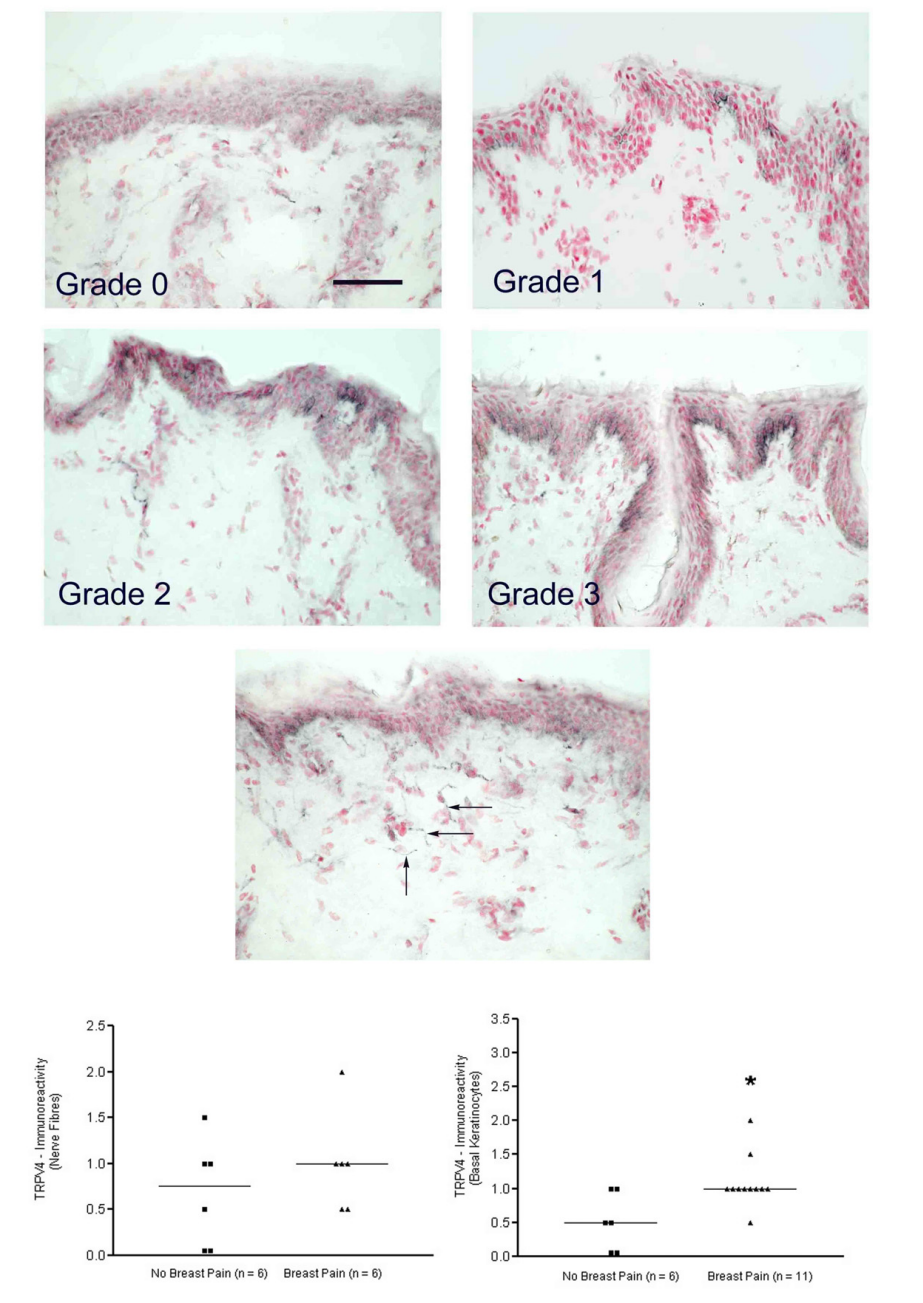
**TRPV4-immunoreactivity in breast skin**. TOP FOUR PANELS: TRPV4-immunoreactivity in keratinocytes mostly at the cell membrane and graded from grade 0/negative (top left) to grade 3/strong staining (bottom right). Scale bar = 100 μm MIDDLE PANEL: Fine, sub-epidermal, TRPV4-immunoreactive fibres (arrows). Scale bar = 100 μm BOTTOM PANELS: Scattergrams showing grading assessment of sub-epidermal fibres (left panel) and keratinocytes (right panel) significantly increased (*P < 0.05) in patients with pain.

Basal keratinocytes also displayed TRPV4 immunoreactivity in groups of cells which were particularly strong at the apex of dermal papillae, where immunoreactivity appeared most strong at the cell membrane junction (Fig 4). Quantification of immunostaining showed a significant (p < 0.03) increase with breast pain (Fig. 4). TRPV4 immunoreactivity was also detected in fine nerve fibres scattered through the sub-epidermis, and showed a trend (p = 0.402) for increased frequency in painful subjects (Fig 4).

#### NGF

**Figure 5:**
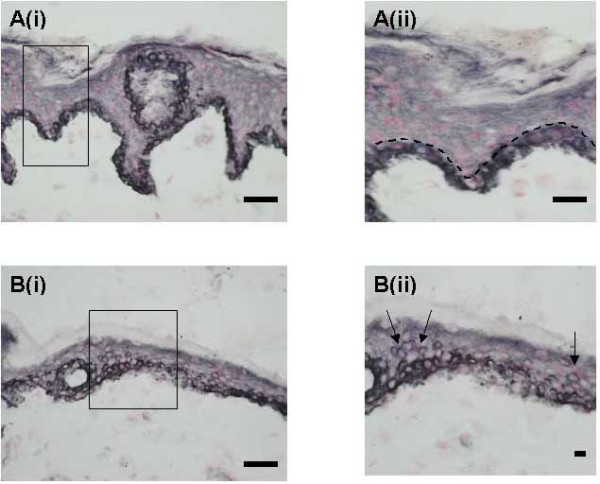
**NGF-immunoreactivity in breast skin**. (**Ai**) Normal, control skin: NGF-immunoreactive basal keratinocytes. (**Aii**)-enlarged area from Ai showing NGF confined to single layer of keratinocytes. (**Bi**) Painful skin (macromastia patient): NGF-immunoreactive basal and supra-basal (**Bii**-arrows) keratinocytes in skin with thin epidermis.

NGF immunoreactivity was present in basal keratinocytes in all samples (Fig 5Ai, ii) with little difference in intensity between pain and no pain specimens (Table 1). In some pain specimens the epidermis appeared to be thinner, and there was evidence of NGF expression in suprabasal as well as basal keratinocytes (Fig 5Bi and 5ii, – arrows), which correlated with the presence of TRPV1positive fibres (seen in a serial section from the same case shown in Fig 1B).

#### Nerve marker (neurofilaments)

Neurofilaments showed nerve fibres in sub-epidermal and dermal regions only but no significant changes were detected between groups (Table 1).

## Discussion

While breast pain and tenderness is a common problem, in the majority of women the mechanisms underlying breast pain are poorly understood. Our study focused on Nerve Growth Factor (NGF) and the expression of the capsaicin receptor 1 (TRPV1) in nociceptor fibres, as these are key molecules in pain and hypersensitivity. Our finding, that TRPV1-positive intra-epidermal fibres were significantly increased in patients with breast pain and tenderness, is both novel and important. The increased and abnormal "clusters" of intra-epidermal fibres were shown in patients who had no previous breast surgery, and no known episodes of mastitis. Hence this may be a surrogate marker for "idiopathic" or macromastia related breast pain in some patients. Studies are in progress to correlate changes in TRPV1 nerve fibres with quantitative sensory perception thresholds.

The cause of the increased intra-epidermal fibres is not known. Given the trend to thinning of the epidermis, normally associated with denervation [21], in the biopsies from some patients with breast pain, the increased intra-epidermal fibres here may represent nerve fibre sprouts following cutaneous terminal damage. The intra-epidermal fibre morphology and "clusters" would be in keeping with this explanation, as fibres ran in unusual patterns. There was no overall change in the sub-epidermal fibre counts for TRPV1 or the structural nerve marker neurofilament. NGF-immunostaining intensity was similar in the different groups. However, in some patients, there appeared to be staining for NGF in the supra-basal epidermis in addition to the basal cells, which is usually associated with inflammation or denervation; increased NGF is known to cause collateral sprouting [22]. Further studies, using quantitative NGF assays and in situ hybridisation, need to be performed to address this issue (these studies would require more substantial skin biopsies). The menstrual cycle influences on NGF levels are also difficult to determine with the current sample size.

Our recent studies have demonstrated increased TRPV1-immunoreactive nerve fibres in inflammatory bowel disease [23], and in the mucosal and sub-mucosal layers of patients with rectal hypersensitivity, where they correlated with thermal and mechanical hypersensitivity, suggesting increase of polymodal nociceptors [20]. We proposed that topical capsaicin or resiniferatoxin treatment, which reduces the numbers of TRPV1 positive fibres, may be a useful therapeutic approach in rectal hypersensitivity. Topical capsaicin has been reported to be useful for treatment for post-mastectomy chronic pain [24], but it is uncertain if it could substantially help breast pain or tenderness in macromastia, as some of the symptoms are likely to arise from deeper structures. Oral selective TRPV1 antagonists thus deserve consideration: both mechanical and thermal hyperalgesia may be reversed by capsazepine, a TRPV1 antagonist [25], again suggesting an effect on polymodal nociceptors. Thus patients may be helped with respect to mechanical symptoms, which predominate in comparison with thermal descriptors.

Little is known of the roles of TRPV3 and TRPV4 in human pain pathophysiology and keratinocyte function. While we have previously demonstrated TRPV3 in human sensory neurons [18], no TRPV3 staining was observed in skin innervation in this study, presumably as the levels in the periphery were below the detection limit of our method. It may be speculated that increased TRPV3 and TRPV4 in observed in keratinocytes may alter keratinocyte expression of NGF and other molecules, which in turn may sensitise nociceptors.

## Conclusion

Breast pain and tenderness appears to be associated with abnormal intra-epidermal innervation. This may reflect re-innervation of skin following nerve stretch damage, and/or collateral sprouting. While further studies are necessary to establish functional links between the TRPV1, TRPV3 and TRPV4 immunohistological changes and breast pain, our findings indicate a path for increasing understanding and treatment of breast pain.

## List of abbreviations

TRPV = transient receptor potential vanilloid; NGF = Nerve Growth Factor; VAS = Visual Analogue Score; PRI = Pain Rating Index

## Competing interests

The author(s) declare that they have no competing interests.

## Authors' contributions

PG and EW recruited patients, collected biopsies and participated in immunohistology studies. PF participated in immunohistology and coordination of the study. AH participated in design of the study and recruitment of patients. JD, GS and CB provided antibodies and helped draft the manuscript. PA conceived the original study, its design and coordination, and helped with the manuscript. All authors read and approved the final manuscript.

## Pre-publication history

The pre-publication history for this paper can be accessed here:


